# Novel ABCB4 mutation in a Chinese female patient with progressive familial intrahepatic cholestasis type 3: a case report

**DOI:** 10.1186/s13000-020-00955-7

**Published:** 2020-04-22

**Authors:** Zhenping Wu, Siying Zhang, Lunli Zhang, Ming Li

**Affiliations:** 1 Department of Critical Care Medicine, The First Affiliated Hospital, College of Medicine, Zhejiang University, Hangzhou, Zhejiang China; 2 Department of Radiology, The First Affiliated Hospital, College of Medicine, Zhejiang University, Hangzhou, Zhejiang China; 3 Department of Infectious Diseases, The First Affiliated Hospital of Nanchang University, Nanchang, Jiangxi China

**Keywords:** *ABCB4*, Progressive familial intrahepatic cholestasis type 3, Novel mutation, Multidrug-resistant protein 3

## Abstract

**Background:**

Progressive familial intrahepatic cholestasis (PFIC) is a rare group of autosomal recessive hereditary hepatic diseases. There are three types of PFIC, classified according to the mutated gene. For example, PFIC type 3 (PFIC3) is due to mutations in the *ABCB4* gene (encoding multidrug-resistant protein 3 [MDR3]).

**Case presentation:**

We present a 19-year-old Chinese female patient who had a 2-year history of recurrent liver dysfunction, with mainly elevated alkaline phosphatase and γ-glutamyl transpeptidase(γ-GT) levels. After excluding other causes of abnormal liver function and cholestasis, the final diagnosis of PFIC3 was confirmed by histopathological examination and gene detection. The immunohistochemical results showed no MDR3 protein expression in the bile duct membrane. Genetic sequencing analysis revealed a novel heterozygous 2137G > A; p. V713M mutation (Exon 17) and a synonymous 504C > T; p. N168N mutation (Exon 6) in *ABCB4*.

**Conclusions:**

Our patient with long-term liver dysfunction demonstrated that elevated alkaline phosphatase and γ-GT levels should be associated with the diagnosis of PFIC3, and gene detection is the key to diagnosis. From our in silico analysis, the novel mutation p. V713M in Exon 17 was predicted to affect protein function, with a SIFT (Sorting Intolerant from Tolerant) score of 0.02, indicating a deleterious effect. Further studies are necessary to investigate the impact of the novel heterozygous 2137G > A; p. V713M mutation (Exon 17) on functional defects of MDR3 and PFIC3.

## Background

Progressive familial intrahepatic cholestasis (PFIC) is a rare group of autosomal recessive hereditary hepatic diseases. Gene mutations result in bile secretion or excretion disorders, and the main clinical manifestation of patients is intrahepatic cholestasis. Without intervention, patients would progress to liver cirrhosis or failure eventually [1]. There are three types of PFIC, classified according to the mutated gene as follows: PFIC type 1 (PFIC1) is due to mutations in the *ATP8B1* gene (encoding FIC1); PFIC type 2 (PFIC2), mutations in the *ABCB11* gene (encoding bile salt export pump); and PFIC type 3 (PFIC3), mutations in the *ABCB4* gene (encoding multidrug-resistant protein 3 [MDR3]). Patients with PFIC1 and PFIC2 have normal or low serum γ-GT levels, but patients with PFIC3 are characterized by high serum γ-GT levels [2, 3]. Patients with PFIC3 usually develop cholestasis in late infancy (one third of cases) to adolescent age group. Gastrointestinal bleeding due to cirrhosis and portal hypertension may be the first presentation in older children or young adults. The disease usually progresses from chronic cholestasis with or without jaundice to portal hypertension and end stage liver disease [3].

We present the case of a 19-year-old female patient who had a 2-year history of recurrent liver dysfunction, with mainly elevated alkaline phosphatase andγ-GT levels. After excluding other causes of abnormal liver function and cholestasis, the final diagnosis of PFIC3 was confirmed by gene detection. Genetic sequencing analysis revealed a novel heterozygous 2137G > A; p. V713M mutation (Exon 17) and a synonymous 504C > T; p. N168N mutation (Exon 6) in *ABCB4*.

## Case presentation

A 19-year-old female patient, a high school student, presented with abnormal liver function with unknown etiology for 2 years and was admitted to our hospital on October 17, 2018. Two years before, she was found to have abnormal liver function with no symptom in a physical examination but with mainly elevated alkaline phosphatase and γ-GT levels (Table 1). Her liver function improved slightly after taking some liver-protective drugs (Ursodeoxycholic Acid and Bicyclol) at the outpatient department. The subsequent follow-up liver function tests still yielded abnormal results; thus, we suggested that the patient be hospitalized to identify the causes of her abnormal liver function. However, because she was asymptomatic and busy in her senior high school year, she refused hospitalization. She revisited our hospital outpatient clinic again on October 17, 2018. This time, she was admitted to our hospital, because liver ultrasonography revealed cirrhosis.

**Table 1:** Liver function throughout the course of the disease

	2 years ago	1 years ago	Admission	Discharge
ALT(U/L)	80	53	62	55
AST(U/L)	65	36	45	43
TBil (μmol/L)	15.8	12.4	14.9	15.0
DBil (μmol/L)	7.2	5.8	4.7	4.5
ALP(U/L)	623	465	300	260
γ-GT (U/L)	365	255	119	102
ALB(g/L)	46.3	40.1	43.9	41.0

*ALT* alanine aminotransferase; *AST* aspartate aminotransferase; *ALP* alkaline phosphatase; *γ-GT* γ-glutamyl transpeptidase; *ALB* albumin; *TBil* total bilirubin; *DBil* direct bilirubin

**Fig. 1:**
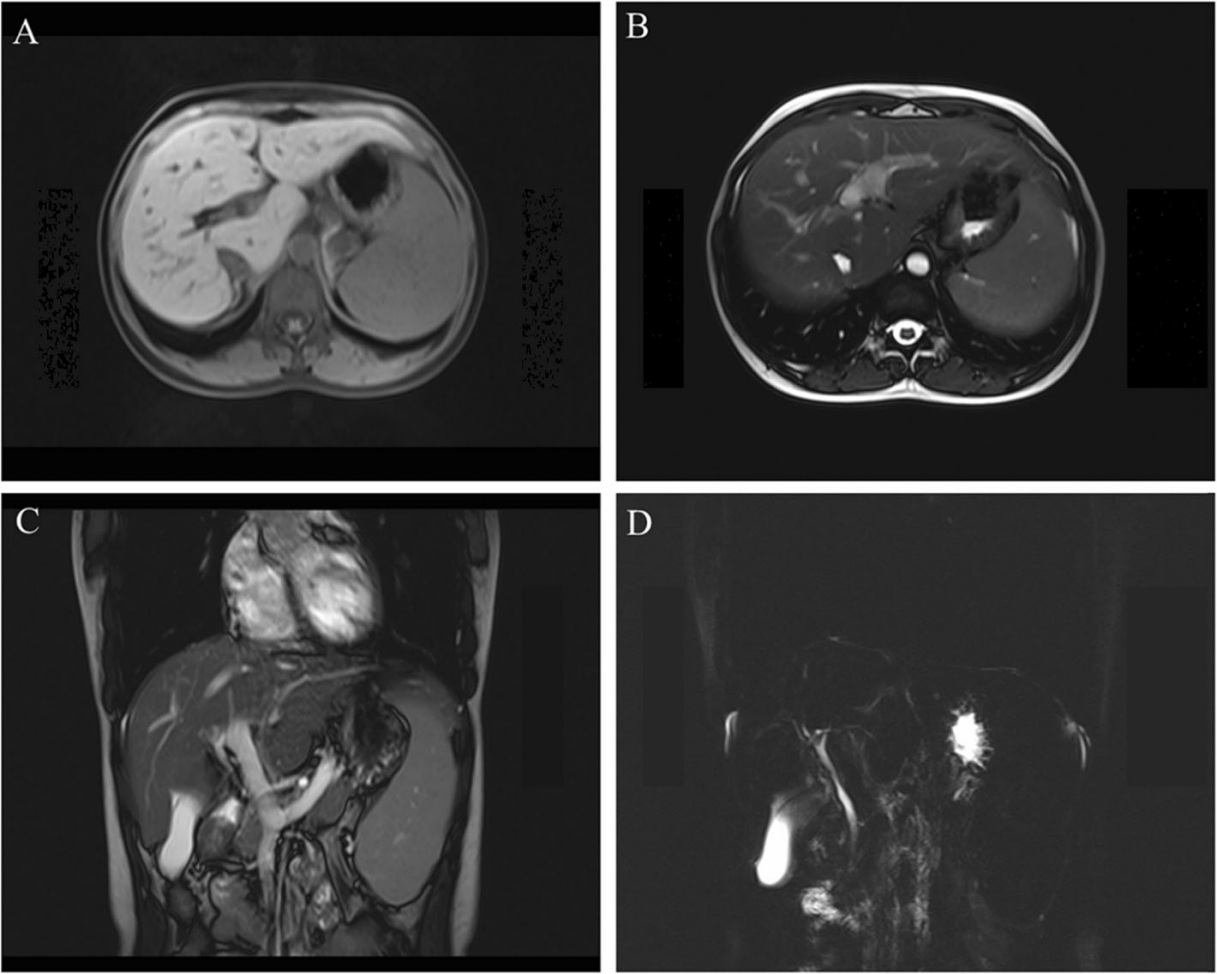
Liver magnetic resonance image. The abdominal magnetic resonance image shows liver cirrhosis, portal hypertension, splenomegaly, and cholestasis. No obstruction of the intrahepatic and extrahepatic bile ducts was found after further magnetic resonance cholangiopancreatographic examination

**Fig. 2:**
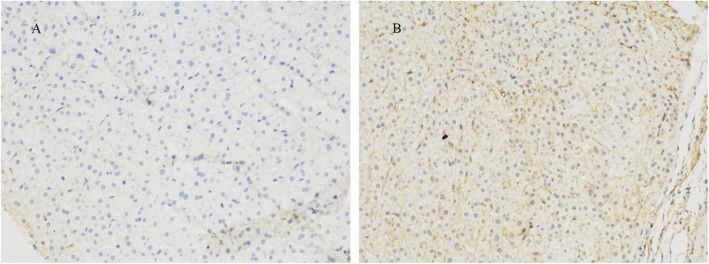
Expression of the multidrug-resistant protein 3 in liver biopsy specimens. **a**: Liver biopsy specimens from the patient: MDR3 protein staining decreased significantly (immunohistochemical staining; original magnification × 200). **b**: Liver biopsy specimens from a healthy person showing strong staining and normal expression of the MDR3 protein (immunohistochemical staining; original magnification × 200)

After admission to our department, we found that the patient had no jaundice, pruritus, asthenia, anorexia, and other discomforts. She denied a history of alcohol intake and use of any hepatotoxic drugs. She also had no family history of liver disease. On physical examination, mild splenomegaly was observed. The results of the rest of the examinations were unremarkable. Her laboratory data (October 18, 2018) showed the following values: alanine aminotransferase, 62 U/L; aspartate aminotransferase 45 U/L; alkaline phosphatase, 300 U/L; γ-GT, 119 U/L; total bilirubin, 14.9 μmol/L; direct bilirubin, 4.7 μmol/L; and albumin, 43.9 g/L. The hepatitis virus markers (hepatitis A, B, C, and E) were negative. Antibodies against the cytomegalovirus and Epstein-Barr virus were also negative. Her ceruloplasmin and serum copper levels were normal, and no Kayser-Fleischer ring was observed upon examination by an experienced ophthalmologist. The qualitative urinary porphyrin test result was negative. All autoimmune antibodies, including antimitochondrial, antinuclear, and antineutrophil cytoplasmic antibodies, were negative. The serum α-1-antitrypsin concentration, thyroid function, coagulation function, and other laboratory investigation results were normal. Abdominal magnetic resonance imaging revealed liver cirrhosis, portal hypertension, splenomegaly, and cholestasis. No obstructions of the intrahepatic and extrahepatic bile ducts were found after further examination with magnetic resonance cholangiopancreatography (Fig. 1). With the consent of the patient, we performed a liver pathological examination. Histological analysis revealed that the MDR3 protein staining decreased significantly as compared with that in healthy persons (Fig. 2).

Combined with the patient’s medical history and results of related tests, we considered the possibility of PFIC3. With her consent, we performed gene mutation detection. Genomic DNA was purified from peripheral blood samples. All 27 coding exons of *ABCB4*, together with at least 100 bp of the adjacent intronic sequence, were amplified by polymerase chain reaction and directly sequenced. The result revealed a synonymous mutation on Exon 6 (c.504C > T; p.N168N; heterozygous) and a novel missense mutation on Exon 17 (c.2137G > A; p.V713M; heterozygous). Based on these data, a diagnosis of PFIC3 was made.

After finding the cause of the disease, the patient was treated with ursodeoxycholic acid 750 mg/d. Considering that the patient had progressed to cirrhosis, we suggested that she should be treated with partial external biliary diversion or liver transplantation. However, her family rejected our suggestion because of the high cost, so she was discharged on November 11, 2018. The liver function test results during the course of the disease are shown in Table 1.

## Discussion

PFIC3 is a rare disease, and most cases are sporadic. Hence, the incidence of the disease has not been accurately reported. A survey showed that the mean incidence of PFIC3 was 1 in 500,000 people [4]. In China, in our literature search, reports of such cases in last 10 years are limited [5–7]. Increasing the coverage of PFIC3 is meaningful and thus can improve the current understanding of this disease.

In this study, we present the case of a 19-year-old Chinese female patient with a novel mutation in *ABCB4* who experienced intrahepatic cholestasis of unknown etiology 2 years before. After excluding other etiologies such as biliary atresia, sclerosing cholangitis, Alagille syndrome, primary biliary cirrhosis, and drug-induced liver injury by laboratory, radiographic, and histological examinations, we highly suspected that this disease might be PFIC3 according to her high γ-GT level. Thus, we performed a gene mutation analysis, which revealed a novel missense heterozygous 2137G > A; p. V713M mutation and a synonymous 504C > T; p. N168N mutation in *ABCB4*. On the basis of these data, the patient was diagnosed as having PFIC3.

PFIC3 is a disease caused by a genetic defect in the *ABCB4* gene encoding the MDR3 protein, which is located on the canalicular membrane of the hepatocyte. The MDR3 protein acts as a phospholipid translocator that transfers phospholipids from hepatocytes to the bile ducts, which is the rate-limiting step of phospholipid secretion in the bile duct [4]. Normally, the phospholipids synthesized by hepatocytes are transported to the bile by MDR3, and phospholipids combine with bile salts to form microparticles, thereby increasing the hydrophilicity of bile salts, reducing the detergency of bile salts, and protecting the cholangiocytes from the bile salts [8]. Mutations in the *ABCB*4 gene lead to the decrease in the secretion of the phospholipid translocator MDR3 protein. The phospholipid in the bile is deficient, and the mixture of bile salts and phospholipids cannot be formed. The bile canaliculi and biliary epithelium get injured because of continuous exposure to hydrophobic bile salts. The detergent effects of bile salts are no longer being countered by phospholipids, thereby leading to cholangitis, hyperplasia, and inflammatory infiltration of the small bile duct. The disease then gradually progresses to portal area fibrosis, cirrhosis, and liver failure [1, 9].

In our patient, liver immunostaining for MDR3 showed a markedly attenuated, discontinuous, and disordered canalicular staining with apparent spread to the basolateral membrane. This led us to the diagnosis of PFIC3. However, normal liver immunostaining of MDR3 does not preclude a gene defect, as a mutation may induce loss of function but indicate normal synthesis and expression [10]. Thus, a clear diagnosis still requires genetic analysis for *ABCB4*. By using data from the human gene mutation database (http://www.hgmd.org/), we confirmed that > 150 types of diseases are related with the *ABCB4* gene mutations, 50 types of mutations are associated with PFIC3. The mutation types include missense mutation, nonsense mutations, deletion, and small base insert fragments. Illness severity is related to the gene mutation type, and nonsense mutation and deletion easily manifest severe clinical symptoms [6].

The genetic sequencing analysis of *ABCB4* in our patient revealed a synonymous 504C > T; p. N168N mutation and a novel heterozygous 2137G > A; p. V713M mutation. Although synonymous mutations do not change the translated protein products, more and more studies have found that synonymous mutations in germ cells and somatic cells may be harmful, leading to genetic diseases and cancers [11]. The database of harmful synonymous mutations (dbdsm, http://bioinfo.ahu.edu.cn: 8080 / dbdsm / index. JSP) is the first database of harmful synonymous mutations in the human genome. We tried to search this synonymous mutation in the database, and found no 504C > T; p. N168N mutation, however, this synonymous mutation has been reported in patients with intrahepatic cholestasis of pregnancy [12]. The relationship between 504C > T; p. N168N mutation and PFIC3 needs further study.

No other novel heterozygous 2137G > A; p. V713M mutation has been reported yet. To evaluate the functional effects of the novel mutation p. V713M, polymorphism phenotyping v2 (PolyPhen-2) and SIFT (Sorting Intolerant from Tolerant) were used. PolyPhen-2 is a tool that predicts the possible impact of an amino acid substitution on the structure and function of a human protein by using straightforward physical and comparative considerations. SIFT predicts whether an amino acid substitution affects protein function. SIFT prediction is based on the degree of conservation of amino acid residues in sequence alignments derived from closely related sequences, collected through PSI-BLAST. SIFT predicts substitutions with scores of < 0.05 as deleterious. Mutation p. V713M is predicted to be damaging at a score of 0.995 (sensitivity: 0.68; specificity: 0.97) by PolyPhen-2. SIFT scores showed that the novel mutation p. V713M was predicted to affect protein function at a SIFT score of 0.02, which indicates a deleterious effect.

## Conclusions

Our patient with long-term liver dysfunction had elevated alkaline phosphatase and γ-GT levels, which should be associated with the diagnosis of PFIC3. Gene detection is the key to diagnosis. From our in silico analysis, we predicted that the novel mutation p. V713M in Exon 17 affects protein function at a SIFT score of 0.02, which indicates a deleterious effect. Further studies are necessary to investigate the impact of the novel heterozygous 2137G > A; p. V713M mutation (Exon 17) on the functional defects of MDR3 and PFIC3.

## Data Availability

The datasets used and/or analyzed in this study are available from the corresponding author on reasonable request.

## Abbreviations

PFIC: Progressive familial intrahepatic cholestasis
MDR3: Multidrug-resistant protein 3
γ-GT: γ-glutamyl transpeptidase
ICP: Intrahepatic cholestasis of pregnancy
